# Immunogenicity and safety of the *Haemophilus influenzae* type b and *Neisseria meningitidis* serogroups C and Y-tetanus toxoid conjugate vaccine co-administered with human rotavirus, hepatitis A and 13-valent pneumococcal conjugate vaccines: results from a phase III, randomized, multicenter study in infants

**DOI:** 10.1080/21645515.2018.1526586

**Published:** 2018-10-05

**Authors:** Nicola P. Klein, Remon Abu-Elyazeed, Yaela Baine, Brigitte Cheuvart, Marcela Silerova, Narcisa Mesaros

**Affiliations:** a Kaiser Permanente Vaccine Study Center, Oakland, CA, USA; b GSK, Philadelphia, PA, USA; c GSK, King of Prussia, PA, USA; d GSK, Wavre, Belgium

**Keywords:** *Haemophilus influenzae* type b, *Neisseria meningitidis* serogroups C and Y, human rotavirus, hepatitis A, *Streptococcus pneumoniae*, vaccination, co-administration, infant

## Abstract

This phase III, open-label, randomized study (NCT01978093) evaluated the immunogenicity and safety of co-administered *Haemophilus influenzae* type b–*Neisseria meningitidis* serogroups C and Y–tetanus toxoid conjugate vaccine (Hib-MenCY-TT) with human rotavirus vaccine (HRV), hepatitis A vaccine (HAV) and 13-valent pneumococcal conjugate vaccine (PCV13). We randomized 600 infants (1:1) to receive 4 doses of Hib-MenCY-TT at 2, 4, 6 and 12–15 months of age or 3 doses of Hib vaccine conjugated to *N. meningitidis* outer membrane protein complex (Hib-OMP) at 2, 4 and 12–15 months of age. All infants received HRV at 2 and 4 months of age, PCV13 at 2, 4, 6 and 12–15 months of age, HAV at 12–15 and 18–21 months of age, and diphtheria-tetanus-acellular pertussis-hepatitis B-inactivated poliovirus vaccine at 2, 4 and 6 months of age. We measured immune responses against HRV, HAV and Hib with enzyme-linked immunosorbent assays, and against MenC/MenY with serum bactericidal assays using human complement. The 4-dose vaccination series with Hib-MenCY-TT induced a robust immune response against Hib, which was non-inferior to that induced by a 3-dose vaccination series with Hib-OMP, and against MenC and MenY. Hib-MenCY-TT did not interfere with immune responses to concomitantly administered HRV, PCV13 and HAV. We did not identify any safety concern. In conclusion, we showed that 4-dose vaccination series with Hib-MenCY-TT during infancy did not interfere with immune responses of co-administered HRV, PCV13 and HAV, induced robust immune responses against Hib, MenC and MenY, and had a clinically acceptable safety profile.

## Introduction

Following the introduction of *Haemophilus influenzae* type b (Hib) and pneumococcal conjugate vaccines in the routine infant immunization schedules and the recommendation to vaccinate preteens and adolescents with meningococcal conjugate vaccines in the United States (US), the incidence of bacterial meningitis has declined in the past 20 years.^1-4^ Although *Streptococcus pneumoniae* remains the most common etiologic agent of bacterial meningitis,^5^ *Neisseria meningitidis* is also a leading cause of meningitis in the US, with serogroup B (MenB), serogroup C (MenC) and serogroup Y (MenY) responsible for most cases.^2^

The combined Hib, MenC and MenY tetanus toxoid (TT) conjugate vaccine (Hib-MenCY-TT, *MenHibrix*, GSK) was developed to protect infants against invasive diseases caused by Hib, MenC and MenY without increasing the number of injections in the US immunization schedule and was approved by the Food and Drug Administration in 2012 for use in infants as a 4-dose vaccination series.^6,7^ A previous phase III study showed that a 3-dose primary vaccination series with Hib-MenCY-TT in infants at 2, 4 and 6 months of age followed by a 4th dose at 12–15 months of age had a comparable immunogenicity profile to licensed Hib vaccines, induced a robust immune response against MenC and MenY, and had a clinically acceptable safety profile.^7,8^ In addition, Hib-MenCY-TT did not appear to interfere with the immune responses of the concomitantly administered diphtheria-tetanus-acellular pertussis-hepatitis B-inactivated poliovirus vaccine (DTaP-HBV-IPV), 7-valent pneumococcal conjugate vaccine,^9,10^ mumps-measles-rubella vaccine, and varicella virus vaccine.^11^

This study evaluated the immunogenicity and safety of co-administered Hib-MenCY-TT with the remaining routinely administered concomitant vaccines (human rotavirus vaccine [HRV; *Rotarix*, GSK] and hepatitis A vaccine [HAV; *Havrix*, GSK]) and with the currently available 13-valent pneumococcal conjugate vaccine (PCV13; *Prevnar*, Pfizer).

## Results

### Demographics

**Figure 1.**
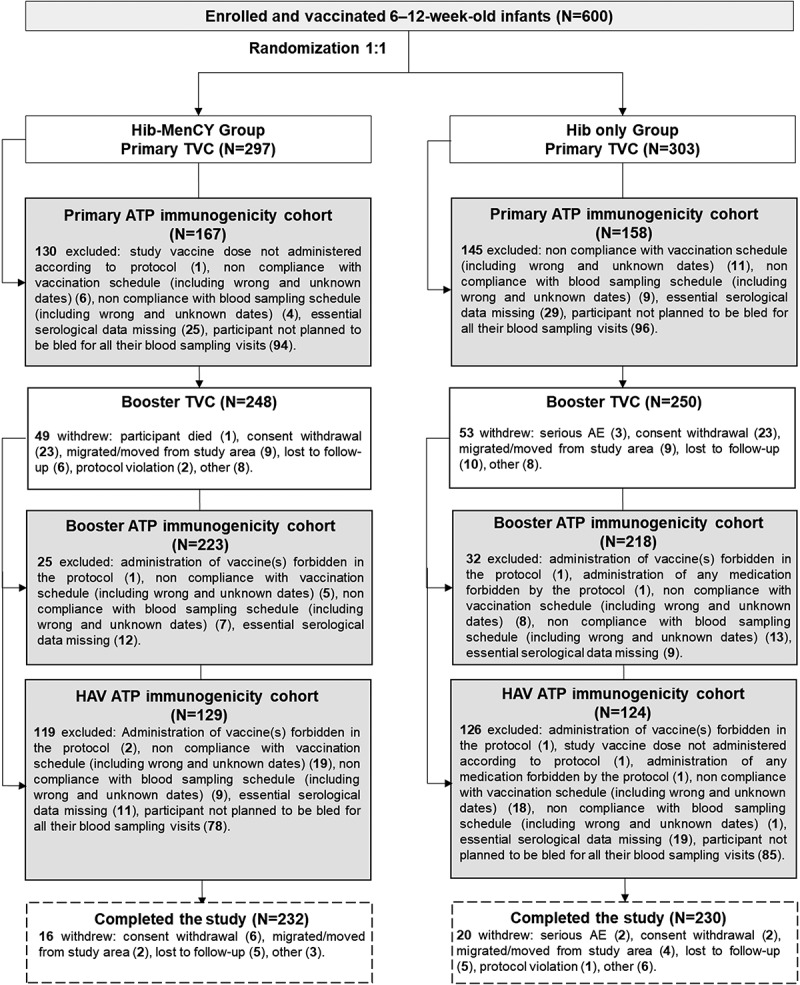
Participant flow chart. ATP, according-to-protocol; TVC, total vaccinated cohort; HAV, hepatitis A vaccine; N, number of participants.

Of the 600 enrolled infants (297 in the Hib-MenCY group and 303 in the Hib only group), 498 were included in the Booster TVC (248 in the Hib-MenCY group and 250 in the Hib only group). The main reason for withdrawal in the primary phase of the study was consent withdrawal. The booster phase of the study was completed by 462 participants (232 in the Hib-MenCY group and 230 in the Hib only group) (Figure 1). During the entire study period, 1 participant in the Hib-MenCY group withdrew due to a fatal SAE (sudden infant death syndrome) and 5 participants in the Hib only group due to SAEs (non-fatal). Eleven participants in the Hib-MenCY group and 15 in the Hib only group were lost to follow-up (Figure 1).

The percentage of African Americans was higher in the Hib-MenCY group than in the Hib only group, especially in the primary according-to-protocol (ATP) immunogenicity cohort (10.8% versus 3.8%). All other demographic characteristics were balanced between the 2 groups in all study cohorts (Table 1, Supplementary Table 1).

**Table 1.** Summary of demographic characteristics (primary and booster TVCs and primary ATP immunogenicity cohorts).

Characteristics	Parameters	Hib-MenCY group	Hib only group
Primary vaccination phase, TVC		N = 297	N = 303
Age at first dose (weeks)	Mean (SD)	8.6 (1.1)	8.6 (1.1)
Gender	Male, n (%)	149 (50.2)	163 (53.8)
Race	White-Caucasian/European Heritage, n (%)	201 (67.7)	218 (71.9)
	African Heritage/African American, n (%)	29 (9.8)	19 (6.3)
	American Indian or Alaskan Native, n (%)	11 (3.7)	12 (4.0)
	Asian – Central/South Asian Heritage, n (%)	5 (1.7)	4 (1.3)
	Asian – East Asian Heritage, n (%)	2 (0.7)	2 (0.7)
	Asian – Japanese Heritage, n (%)	1 (0.3)	0 (0.0)
	Asian – South East Asian Heritage, n (%)	9 (3.0)	8 (2.6)
	Native Hawaiian or other Pacific Islander, n (%)	2 (0.7)	6 (2.0)
	White – Arabic/North African Heritage, n (%)	1 (0.3)	2 (0.7)
	Other, n (%)	36 (12.1)	32 (10.6)
Hepatitis B vaccination at birth	Yes, n (%)	280 (94.3)	291 (96.0)
Primary vaccination phase, ATP immunogenicity cohort		N = 167	N = 158
Age at first dose (weeks)	Mean (SD)	8.7 (1.2)	8.7 (1.1)
Gender	Male, n (%)	85 (50.9)	82 (51.9)
Race	White-Caucasian/European Heritage, n (%)	109 (65.3)	114 (72.2)
	African Heritage/African American, n (%)	18 (10.8)	6 (3.8)
	American Indian or Alaskan Native, n (%)	7 (4.2)	8 (5.1)
	Asian – Central/South Asian Heritage, n (%)	2 (1.2)	2 (1.3)
	Asian – East Asian Heritage, n (%)	1 (0.6)	2 (1.3)
	Asian – Japanese Heritage, n (%)	1 (0.6)	0 (0.0)
	Asian – South East Asian Heritage, n (%)	5 (3.0)	5 (3.2)
	Native Hawaiian or other Pacific Islander, n (%)	2 (1.2)	3 (1.9)
	White – Arabic/North African Heritage, n (%)	1 (0.6)	0 (0.0)
	Other, n (%)	21 (12.6)	18 (11.4)
Hepatitis B vaccination at birth	Yes, n (%)	156 (93.4)	154 (97.5)
Booster vaccination phase, TVC		N = 248	N = 250
Age at 4th dose (months)	Mean (SD)	12.5 (0.8)	12.6 (0.8)
Gender	Male, n (%)	124 (50.0)	135 (54.0)
Race	White-Caucasian/European Heritage, n (%)	174 (70.2)	183 (73.2)
	African Heritage/African American, n (%)	21 (8.5)	13 (5.2)
	American Indian or Alaskan Native, n (%)	8 (3.2)	10 (4.0)
	Asian – Central/South Asian Heritage, n (%)	4 (1.6)	3 (1.2)
	Asian – East Asian Heritage, n (%)	2 (0.8)	0 (0.0)
	Asian – Japanese Heritage, n (%)	1 (0.4)	0 (0.0)
	Asian – South East Asian Heritage, n (%)	7 (2.8)	8 (3.2)
	Native Hawaiian or other Pacific Islander, n (%)	2 (0.8)	4 (1.6)
	White – Arabic/North African Heritage, n (%)	1 (0.4)	2 (0.8)
	Other, n (%)	28 (11.3)	27 (10.8)
Hepatitis B vaccination at birth	Yes, n (%)	236 (95.2)	240 (96.0)

N, number of participants; SD, standard deviation, n (%), number (percentage) of participants in a given category, ATP, according-to-protocol; TVC, total vaccinated cohort

### Immunogenicity

#### Non-inferiority criteria

Protocol pre-specified statistical criteria for non-inferiority were met for all co-primary objectives (Table 2). Non-inferiority of 4 doses of Hib-MenCY compared with 3 doses of Hib vaccine conjugated to *N. meningitidis* outer membrane protein complex (Hib-OMP; *PedvaxHIB*, Merck), each co-administered with PCV13 and HAV, was demonstrated in terms of concentrations of antibodies (≥ 1.0 µg/mL) against the capsular Hib polysaccharide polyribosylribitol phosphate (PRP) .

**Table 2.** Summary of the co-primary objectives (primary, booster and HAV ATP immunogenicity cohorts).

Evaluation		Results	
Objective	Statistical criterion	Assessed outcome	Value (95% CI)
1. To demonstrate the non-inferiority of a 4-dose vaccination course with Hib-MenCY-TT compared with a 3-dose vaccination course with Hib-OMP, each co-administered with PCV13 and HAV, in terms of anti-PRP antibody concentration ≥ 1.0 µg/mL.^a^	One month post-dose 4: LL of standardized asymptotic 95% CI for the difference in % of participants with anti-PRP antibody concentration ≥ 1.0 µg/mL between the two groups ≥-10%.	Difference between groups (anti-PRP antibody concentration): Hib-MenCY minus Hib only group	0.96 (−2.**12**; 4.30)
**Primary vaccination phase**			
**Value (97.5% CI)**			
2. To demonstrate the non-inferiority of a 2-dose primary vaccination course with HRV co-administered with Hib-MenCY-TT, DTaP-HBV-IPV and PCV13 compared with that of HRV co-administered with Hib-OMP, DTaP-HBV-IPV and PCV13 in terms of anti-HRV IgA GMCs.	Two months post-dose 2: LL of 97.5% CI of anti-HRV IgA GMC ratio ≥ 0.5.	Anti-HRV IgA GMC ratio between groups: Hib-MenCY group/Hib only group	1.21 (**0.77**; 1.90)
3. To demonstrate the non-inferiority of a 3-dose primary vaccination course of PCV13 co-administered with Hib-MenCY-TT, HRV and DTaP-HBV-IPV compared with that of PCV13 co-administered with Hib-OMP, HRV and DTaP-HBV-IPV in terms of anti-pneumococcal antibody GMCs.	One month post-dose 3: LL of 97.5% CI of anti-pneumococcal serotypes 1, 3, 4, 5, 6A, 6B, 7F, 9V, 14, 18C, 19A, 19F and 23F antibody GMC ratio ≥ 0.5.	Anti-pneumococcal antibody GMC ratio between groups: Hib-MenCY group/Hib only group	anti-1, 1.18 (**0.95**; 1.47)
			anti-3, 1.15 (**0.93**; 1.42)
			anti-4, 1.08 (**0.90**; 1.31)
			anti-5, 1.18 (**0.95**; 1.47)
			anti-6A 1.29 (**1.03**; 1.63)
			anti-6B 1.17 (**0.88**; 1.55)
			anit-7F, 1.11 (**0.91**; 1.34)
			anti-9V, 1.25 (**1.00**; 1.55)
			anti-14, 1.16 (**0.90**; 1.50)
			anti-18C, 1.24 (**1.01**; 1.52)
			anti-19A, 1.16 (**0.94**, 1.43)
			anti-19F, 1.07 (**0.89**; 1.29)
			anti-23F, 1.18 (**0.91**; 1.53)
**Booster vaccination phase**			
4. To demonstrate the non-inferiority of a 2-dose vaccination course of HAV when the first dose is co-administered with Hib-MenCY-TT and PCV13 compared with that of HAV when the first dose is co-administered with Hib-OMP and PCV13 in terms of anti-HAV antibody concentration ≥ 15 mIU/mL.	One month after the second HAV vaccination: LL of standardized asymptotic 97.5% CI for the difference in % of participants with antibody concentration ≥ 15 mIU/mL between two groups ≥-10%.	Difference between groups (anti-HAV antibody concentration): Hib-MenCY minus Hib only group	0.00 (−3.**76**; 3.91)
5. To demonstrate the non-inferiority of a 4-dose vaccination course of PCV13 co-administered with Hib-MenCY-TT and HAV compared with that of PCV13 co-administered with Hib-OMP and HAV in terms of anti-pneumococcal antibody GMCs.	One month post-dose 4: LL of 97.5% CI of anti-pneumococcal serotypes 1, 3, 4, 5, 6A, 6B, 7F, 9V, 14, 18C, 19A, 19F and 23F antibody GMC ratio ≥ 0.5.	Anti-pneumococcal antibody GMC ratio between groups: Hib-MenCY group/Hib only group	anti-1, 1.25 (**1.04**; 1.51)
			anti-3, 1.01 (**0.83**; 1.24)
			anti-4, 1.10 (**0.92**; 1.31)
			anti-5, 1.06 (**0.87**; 1.28)
			anti-6A 1.21 (**1.01**; 1.44)
			anti-6B 1.13 (**0.94**; 1.36)
			anit-7F, 1.09 (**0.93**; 1.29)
			anti-9V, 1.12 (**0.94**; 1.33)
			anti-14, 1.16 (**0.96**; 1.41)
			anti-18C, 1.14 (**0.97**; 1.35)
			anti-19A, 1.09 (**0.90**, 1.31)
			anti-19F, 1.12 (**0.95**; 1.34)
			anti-23F, 1.22 (**1.00**; 1.50)

ATP, according-to-protocol; CI, confidence interval; IgA, immunoglobulin A; LL, lower limit; GMC, geometric mean concentration; Hib-MenCY-TT, *Haemophilus influenzae* type b and *Neisseria meningitidis* serogroups C and Y-tetanus toxoid conjugate vaccine; Hib-OMP, Hib vaccine conjugated to *N. meningitidis* outer membrane protein complex; HRV, human rotavirus vaccine; PCV13, 13-valent pneumococcal conjugate vaccine; DTaP-HBV-IPV, diphtheria-tetanus-acellular pertussis-hepatitis B surface antigen-inactivated poliovirus vaccine; HAV, hepatitis A vaccine.

^a^ As per hierarchical procedure, the statistical criteria for the first objective needed to be met before any objective of the primary or booster vaccination phase could be met. Bold values indicate the objective was met.

In the primary vaccination phase, non-inferiority of 2 primary doses of HRV co-administered with Hib-MenCY-TT, DTaP-HBV-IPV and PCV13 compared with that of HRV co-administered with Hib-OMP, DTaP-HBV-IPV and PCV13 was demonstrated in terms of anti-HRV immunoglobulin A (IgA) geometric mean concentrations (GMCs). Non-inferiority of 3 primary doses of PCV13 co-administered with Hib-MenCY-TT, DTaP-HBV-IPV and HRV compared with that of PCV13 co-administered with Hib-OMP, DTaP-HBV-IPV and HRV was demonstrated in terms of anti-pneumococcal serotypes 1, 3, 4, 5, 6A, 6B, 7F, 9V, 14, 18C, 19A, 19F and 23F antibody GMCs (Table 2).

In the booster vaccination phase, non-inferiority of 2 doses of HAV when the first dose was co-administered with Hib-MenCY-TT and PCV13 compared with that of HAV when the first dose was co-administered with Hib-OMP and PCV13 was demonstrated in terms of anti-HAV antibody concentrations ≥ 15 mIU/mL. Non-inferiority of 4 doses of PCV13 co-administered with Hib-MenCY-TT and HAV compared with that of PCV13 co-administered with Hib-OMP and HAV was demonstrated in terms of anti-pneumococcal serotypes 1, 3, 4, 5, 6A, 6B, 7F, 9V, 14, 18C, 19A, 19F and 23F antibody GMCs (Table 2).

The results of the additional analyses performed on the total vaccinated cohorts (TVCs) were similar to those obtained on the ATP immunogenicity cohorts for all the co-primary objectives (Supplementary Table 2).

#### Primary immune responses

In the primary ATP immunogenicity cohort, 94.0% of participants had anti-PRP antibody concentrations ≥ 1 µg/mL at 1 month post-dose 3 in the Hib-MenCY group and 91.5% at 2 months post-dose 2 in the Hib only group (Supplementary Table 3). Anti-PRP antibody GMCs were 8.414 µg/mL in the Hib-MenCY group and 11.053 µg/mL in the Hib only group.

Two months post-dose 2, ≥ 80.1% of participants had anti-HRV IgA concentrations ≥ 20 U/mL in both groups (Supplementary Table 3). Anti-HRV IgA GMCs were comparable between groups (138.9 U/mL in the Hib-MenCY group and 115.0 U/mL in the Hib only group).

One month post-dose 3, ≥ 69.3% of participants had anti-pneumococcal antibody concentrations ≥ 0.35 µg/mL against serotype 3, ≥ 76.4% against serotypes 4, 5, 6B, 9V, 18C and 23F, and ≥ 90.5% against serotypes 1, 6A, 7F, 14, 19A and 19F in the Hib-MenCY and Hib only groups (Supplementary Table 3). Anti-pneumococcal antibody GMCs were similar between groups (based on overlapping 95% confidence intervals [CIs]) and ranged from 0.48 µg/mL (serogroup 3, Hib only group) to 4.77 µg/mL (serogroup 14, Hib-MenCY group).

The percentages of participants with serum bactericidal antibody titers measured using a human complement assay (hSBA) ≥ 1:8 were 100% against MenC and 97.7% against MenY at 1 month after the 3rd Hib-MenCY-TT dose in the Hib-MenCY group, and 1.4% against MenC and 100% against MenY at 3 months after the 2nd Hib-OMP dose in the Hib only group (Supplementary Table 3). hSBA antibody geometric mean titers (GMTs) were 807.3 for MenC and 510.9 for MenY in the Hib-MenCY group, and 2.1 for MenC and 550.2 for MenY in the Hib only group.

#### Booster immune responses

In the booster ATP immunogenicity cohort, ≥ 97.2% of participants had anti-PRP antibody concentrations ≥ 1 µg/mL after the booster dose of Hib-MenCY-TT or Hib-OMP (Supplementary Table 4). Anti-PRP antibody GMCs were 28.090 µg/mL in the Hib-MenCY group and 20.869 µg/mL in the Hib only group.

After the 4th dose of PCV13, ≥ 69.5% of participants had anti-pneumococcal antibody concentrations ≥ 0.35 µg/mL against pneumococcal serotype 3, and ≥ 94.1% against pneumococcal serotypes 1, 4, 5, 6A, 6B, 7F, 9V, 14, 18C, 19A, 19F and 23F (Supplementary Table 4). Anti-pneumococcal antibody GMCs were comparable between groups (based on overlapping 95% CIs), and ranged from 0.51 µg/mL (serogroup 3, Hib only group) to 7.14 µg/mL (serogroup 14, Hib-MenCY group).

In the Hib-MenCY group, ≥ 98.5% of participants had hSBA antibody titers ≥ 1:8 against MenC and MenY after the 4th dose of Hib-MenCY-TT (Supplementary Table 4). In the Hib only group, 0.6% of participants had hSBA antibody titers ≥ 1:8 against MenC and all participants had hSBA antibody titers ≥ 1:8 against MenY after the 3rd dose of Hib-OMP. hSBA GMTs against MenC and MenY were 2566.2 and 2761.4 in the Hib-MenCY group, and 2.0 and 2728.2 in the Hib only group.

#### Immune response to HAV

In both groups, ≥ 85.2% of participants in the booster ATP immunogenicity cohort had anti-HAV antibody concentrations ≥ 15 mIU/mL one month after the first dose of HAV, which increased to 100% of participants in the HAV immunogenicity cohort after the second dose (Supplementary Table 4). Anti-HAV antibody GMCs were 44.8 and 47.3 mIU/mL after the first dose, and increased to 1590.7 and 1390.6 after the second dose in the Hib-MenCY and Hib only groups.

### Safety

**Figure 2.**
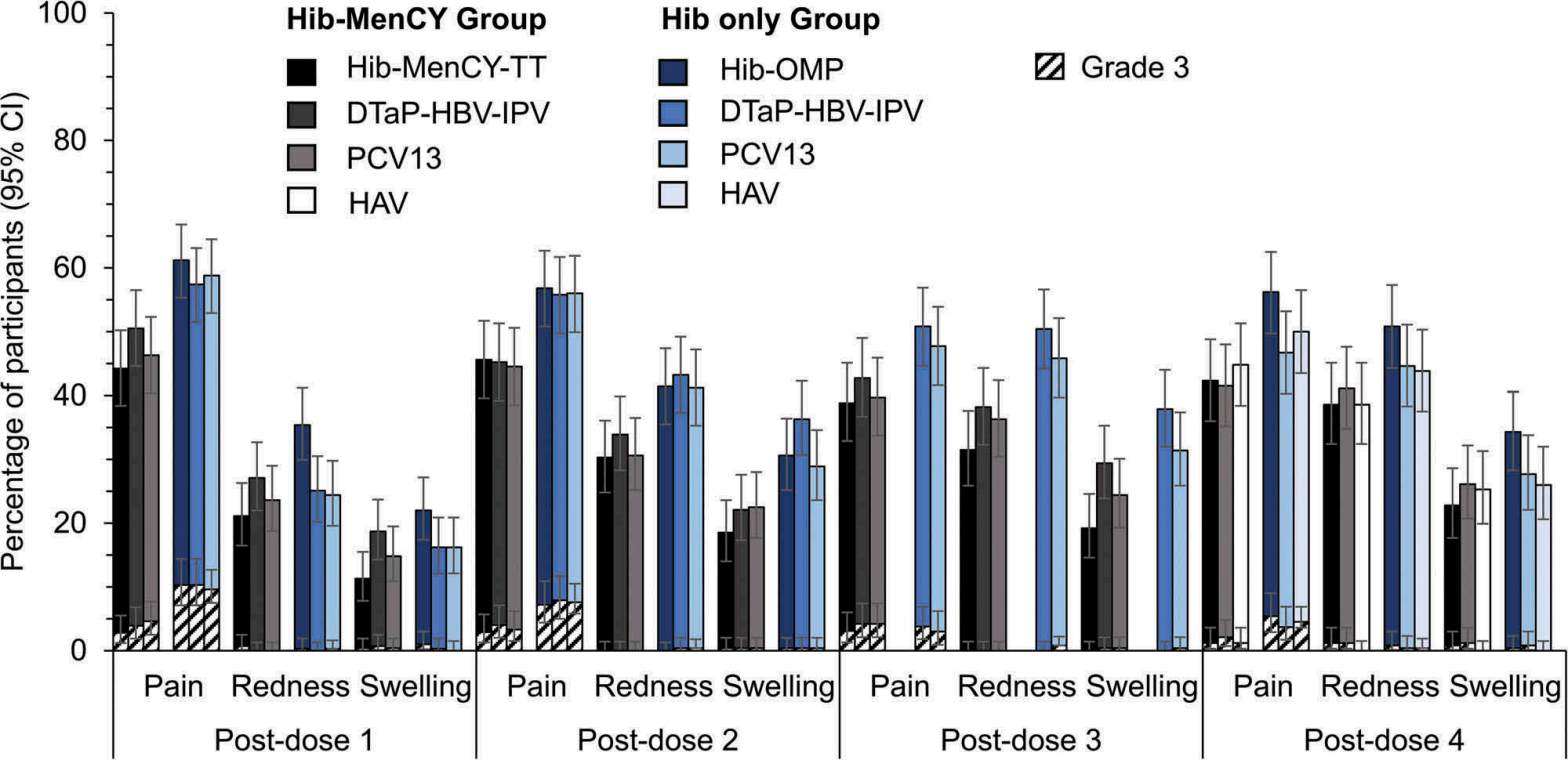
Incidence of solicited local symptoms reported in the 4-day interval following vaccination (primary and booster total vaccinated cohorts). Hib-MenCY-TT, *Haemophilus influenzae* type b and *Neisseria meningitidis* serogroups C and Y-tetanus toxoid conjugate vaccine; Hib-OMP, Hib vaccine conjugated to *N. meningitidis* outer membrane protein complex; PCV13, 13-valent pneumococcal conjugate vaccine; DTaP-HBV-IPV, diphtheria-tetanus-acellular pertussis-hepatitis B surface antigen-inactivated poliovirus vaccine; HAV, hepatitis A vaccine; 95% CI, 95% confidence interval.

The most common solicited local symptoms at the injection site for all co-administered vaccines were pain (38.8%–61.2% after each dose) in the primary TVC, and pain and redness (38.6%–56.2%) in the booster TVC (Figure 2). Across all groups, ≤ 10.3% (primary phase) and ≤ 5.4% (booster phase) of participants reported grade 3 solicited local symptoms at the injection site of each co-administered vaccine.

**Figure 3.**
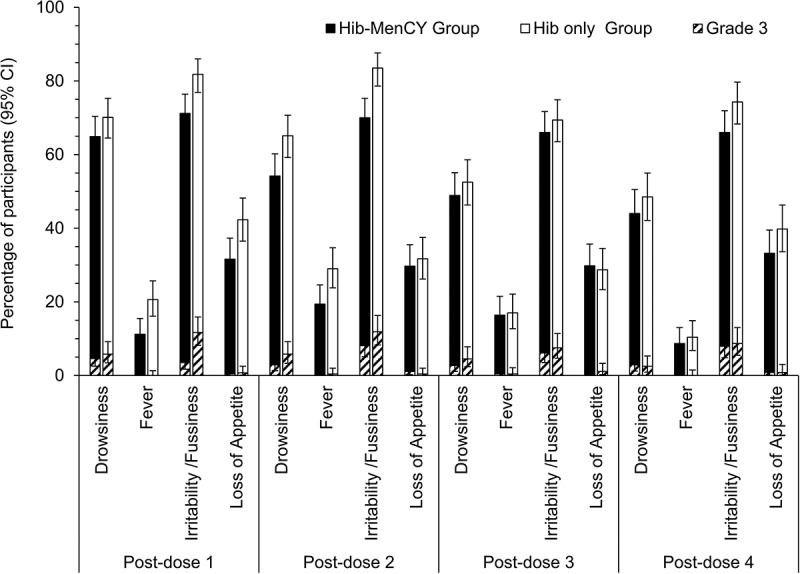
Incidence of solicited general symptoms reported in the 4-day interval following vaccination (primary and booster total vaccinated cohorts). 95% CI, 95% confidence interval.

The most common solicited general symptom in all groups was irritability, reported in 66.0–83.5% of participants after each primary dose and 66.0–74.3% of participants after the booster dose (Figure 3). Across all groups, ≤ 11.9% and ≤ 8.7% of participants reported grade 3 solicited general symptoms after each dose in the primary and booster vaccination phases.

During the 31-day follow-up after each dose and overall in the primary vaccination phase, 180/297 (60.6%) participants in the Hib-MenCY group and 171/303 (56.4%) participants in the Hib only group reported at least one unsolicited adverse event (AE), and 23/297 (7.7%) participants in the Hib-MenCY group and 19/303 (6.3%) participants in the Hib only group reported at least one grade 3 unsolicited AE. The most frequently reported grade 3 unsolicited AEs were upper respiratory tract infections (6/297 participants in the Hib-MenCY and 1/303 participant in the Hib only group) and otitis media (3/297 participants in the Hib-MenCY and 4/303 participants in the Hib only group).

During the 31-day period following the booster dose, 99/248 (39.9%) participants in the Hib-MenCY and 105/250 (42.0%) participants in the Hib only group reported at least one unsolicited AE, and 19/248 (7.7%) participants in the Hib-MenCY and 20/250 (8.0%) participants in the Hib only group reported at least one grade 3 unsolicited AE. The most frequently reported grade 3 unsolicited AEs were otitis media and pyrexia (each in 4/248 participants) in the Hib-MenCY group, and otitis media (5/250 participants) and vomiting (3/250 participants) in the Hib only group.

Up to the day of the booster dose, 5/297 (1.7%) participants in the Hib-MenCY and 10/303 (3.3%) in the Hib only group reported at least one serious AE (SAE). There were 1 case each of gastroesophageal reflux disease, sudden infant death syndrome, gastroenteritis, pneumonia, failure to thrive and apparent life-threatening event in the Hib-MenCY group. The fatal sudden infant death syndrome reported in the Hib-MenCY group was not considered by the investigator as related to vaccination. There were 2 cases of respiratory syncytial virus bronchiolitis, and 1 case each of anemia, hematophagic histiocytosis, neutropenia, hypothermia, subcutaneous abscess, urinary tract infection, viral infection (inconclusive final diagnosis), subdural hematoma, hypernatremia, apparent life-threatening event and Kawasaki disease in the Hib only group.

After the booster dose, 3/248 (1.2%) participants in the Hib-MenCY and 1/250 (0.4%) participants in the Hib only group reported at least one SAE. There were 2 cases of pneumonia, 1 infectious croup cough and 1 accidental overdose (benzodiazepine) in the Hib-MenCY group, and 1 case of ketoacidosis and type 1 diabetes mellitus (same participant) in the Hib only group.

The investigators did not consider any of the SAEs reported during the study period as vaccine-related.

## Discussion

This study evaluated the immunogenicity and safety of the Hib-MenCY-TT vaccine co-administered with HRV, PCV13 and HAV vaccines during infancy. Four doses of the Hib-MenCY-TT vaccine induced a robust immune response against PRP, which was non-inferior to that induced by 3 doses of the Hib-OMP vaccine, and against MenC and MenY. This study also showed that the use of Hib-MenCY-TT vaccine did not interfere with the immune response to the concomitant vaccines. Finally, all co-administered vaccines had a clinically acceptable safety profile.

The robust immune response against PRP induced by Hib-MenCY-TT confirmed the results of previous studies, which also demonstrated the non-inferiority of the immune response to Hib induced by Hib-MenCY-TT over that induced by licensed monovalent Hib vaccines (3 primary doses of Hib-TT followed by a booster dose of Hib-OMP).^8,12^ Further, the higher anti-PRP antibody concentrations after the booster dose of Hib-MenCY-TT compared with those measured after the 3 primary doses confirmed that this vaccine induced immune memory for Hib.^8,12-15^

This study also confirmed that a 3-dose primary vaccination series with Hib-MenCY-TT during infancy followed by a booster dose in the second year of life induced robust immune responses to MenC and MenY.^10,12,13,15^ The 3 primary doses of Hib-MenCY-TT induced high levels of bactericidal antibodies against MenC and MenY in almost all participants, which were consistent with those observed in previous studies.^8,10,12^ The increase in hSBA titers after the 4th dose of Hib-MenCY-TT demonstrates the induction of immune memory for MenC and MenY.^8,12-15^ Unexpectedly, the immune response to MenY after Hib-MenCY-TT vaccination was similar to the immune response to MenY induced by Hib-OMP, a finding which has also been observed to a lesser extent in other studies using Hib-OMP as booster vaccine following three primary doses of Hib-TT.^8,12^ One possible explanation could be that Hib-OMP contains *N. meningitidis* serogroup B outer membrane protein as carrier for the Hib polysaccharide,^8,12^ which produces cross-reactive antibodies to the outer membrane proteins of the MenY target strain used in the hSBA-MenY functional cell killing assay.

Our study also showed that Hib-MenCY-TT did not interfere with the immune responses to the concomitantly administered vaccine, which is in line with the results of previous studies evaluating its co-administration with other routine childhood vaccines.^9-11^ The absence of interference with PCV13 is consistent with a previous study evaluating the co-administration of Hib-MenCY-TT with the 7-valent pneumococcal conjugate vaccine.^9,10^ In our study, the 4th dose of PCV13 did not induce increases in percentages of participants with anti-pneumococcal antibody concentrations ≥ 0.35 µg/mL against serotype 3 compared with the primary vaccination series and percentages of seroprotected participants were lower against serotype 3 than for the other serotypes in both groups, which is in line with findings from a previous study conducted in the US.^16^

This study did not identify any new safety concerns. The safety profile of Hib-MenCY-TT in our study was similar to that observed in previous studies which evaluated its co-administration with the routine recommended vaccines (DTaP-HBV-IPV, 7-valent pneumococcal conjugate vaccine, mumps-measles-rubella vaccine, and varicella virus vaccine).^17^ The safety profiles of HRV, PCV13 and HAV were also similar when co-administered with Hib-MenCY-TT or Hib-OMP, which is also consistent with the results obtained when other routine childhood vaccines were co-administered with Hib-MenCY-TT.^11^

A study limitation was the high drop-out rate observed during the study period as nearly one fourth of participants did not complete the study and nearly half of participants were excluded from the primary ATP immunogenicity cohort, because we had not planned to bleed most of these participants. An additional analysis performed on the TVC suggested that the high drop-out rates did not bias the outcomes of the co-primary objectives, however it should be noted that most of the participants who dropped out did not have immunogenicity results available. Another limitation of this study was its open study design, which might have influenced the safety assessments. Finally, the comparisons between groups should be interpreted with caution because there was no adjustment for multiplicity and the study was only powered for the primary objectives.

In conclusion, we showed that 4 doses of Hib-MenCY-TT co-administered with HRV, PCV13 and HAV during infancy induced a robust immune response against PRP, which was non-inferior to that induced by 3 doses of Hib-OMP vaccine, and against MenC and MenY, and did not interfere with immune responses of the co-administered vaccines. All co-administered vaccines in this study had a clinically acceptable safety profile.

**Figure 4.**
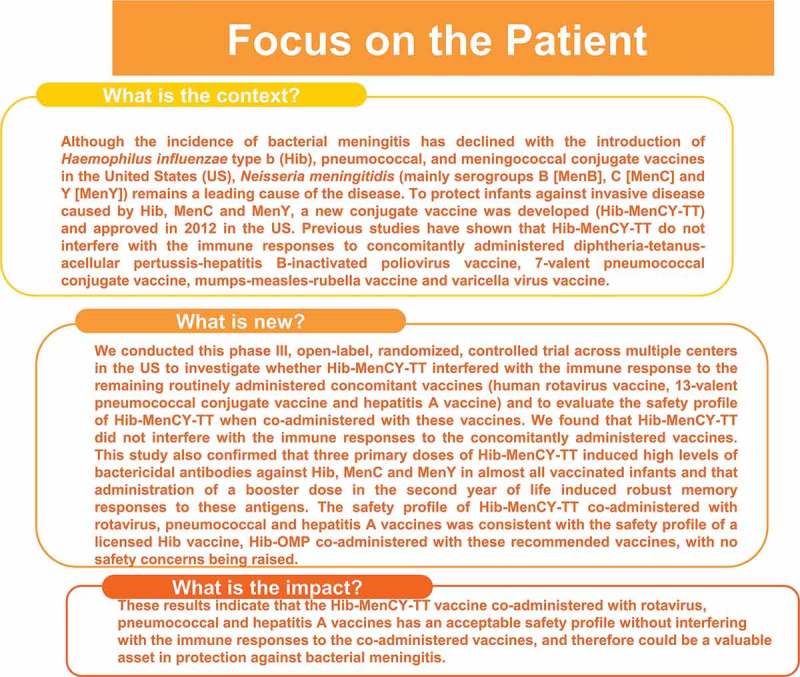
Focus on the patient.

Figure 4 summarizes the research, clinical relevance and impact of this study on the patient population.

## Materials and methods

### Study design and participants

We conducted this phase III, open-label, randomized, controlled trial in 27 centers in the US between February 2014 and March 2016. This study included healthy infants aged 6–12 weeks, who were born after at least 37 weeks of gestation. Supplementary material 1 contains a full list of inclusion/exclusion criteria.

**Figure 5.**
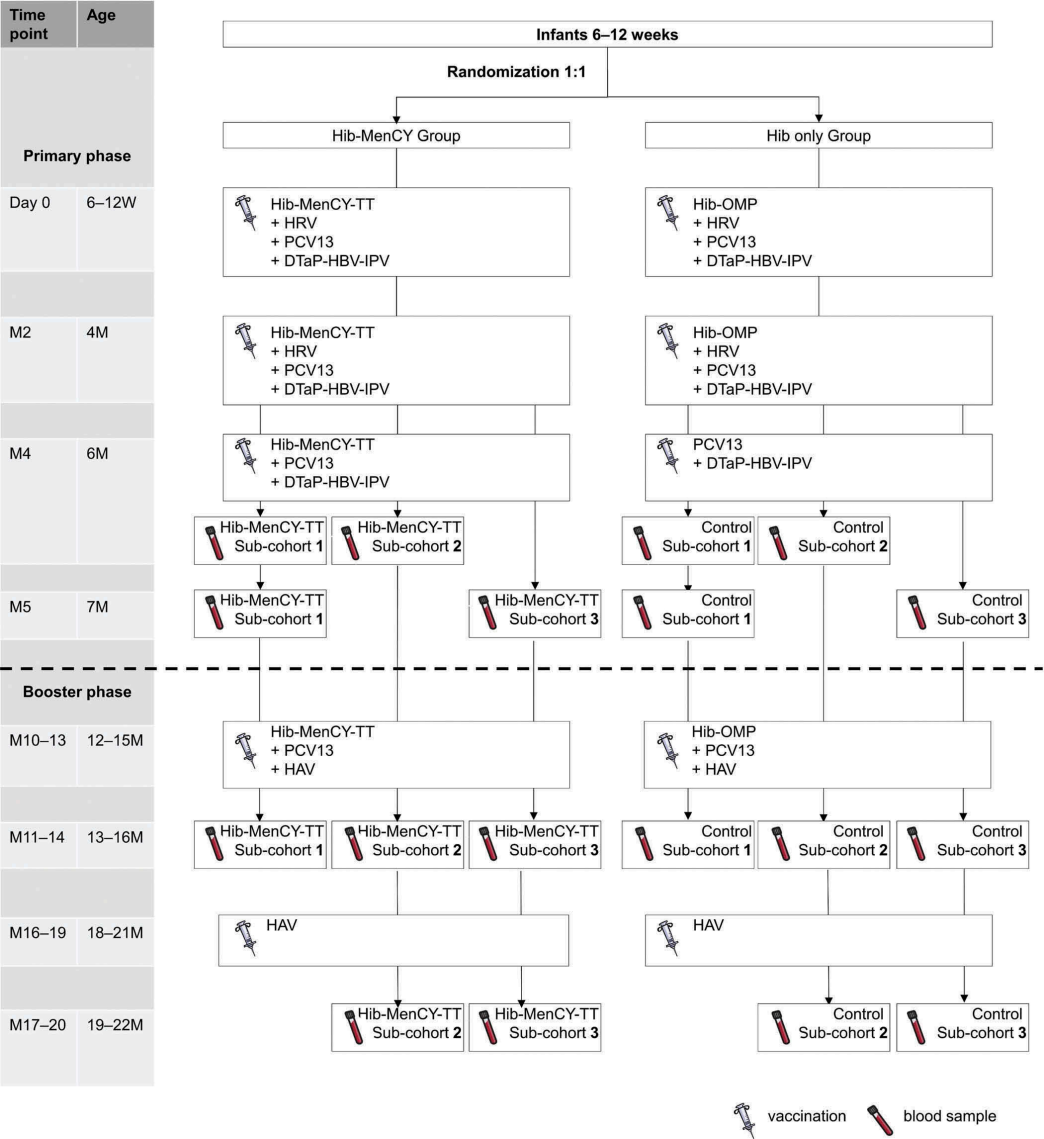
Study design. Hib-MenCY-TT, *Haemophilus influenzae* type b and *Neisseria meningitidis* serogroups C and Y-tetanus toxoid conjugate vaccine; Hib-OMP, Hib vaccine conjugated to *N. meningitidis* outer membrane protein complex; HRV, human rotavirus vaccine; PCV13, 13-valent pneumococcal conjugate vaccine; DTaP-HBV-IPV, diphtheria-tetanus-acellular pertussis-hepatitis B surface antigen-inactivated poliovirus vaccine; HAV, hepatitis A vaccine; M, month; W, WeeksThe first 200 participant were enrolled in the blood sample sub-cohort 3, the next 200 participant were enrolled in the blood sample sub-cohort 2 and the last 200 participant were enrolled in the blood sample sub-cohort 1. An extended safety follow-up was done up to the start of the Booster phase. If a participant did not return for the M10–13 timepoint, the study personnel reviewed the participant’s electronic medical records and/or contacted the participant’s parent/guardian by phone to obtain the safety information.

In order to minimize the number of blood draws in this pediatric population, we enrolled infants into 3 sub-cohorts, each of which provided a blood sample at 3 different timepoints out of the 4 total possible timepoints (Figure 5). Within each sub-cohort, we randomized the infants (1:1) to the Hib-MenCY and Hib only groups. Participants in the Hib-MenCY group received Hib-MenCY-TT co-administered with HRV, PCV13 and DTaP-HBV-IPV at 2 and 4 months of age, Hib-MenCY-TT co-administered with PCV13 and DTaP-HBV-IPV at 6 months of age, a booster dose of Hib-MenCY-TT co-administered with PCV13 and HAV at 12–15 months of age, and HAV at 18–21 months of age. In the Hib only group, participants received Hib-OMP co-administered with HRV, PCV13 and DTaP-HBV-IPV at 2 and 4 months of age, PCV13 co-administered with DTaP-HBV-IPV at 6 months of age, a booster dose of Hib-OMP co-administered with PCV13 and HAV at 12–15 months of age, and HAV at 18–21 months of age.

We administered HRV orally and all other vaccines intramuscularly (DTaP-HBV-IPV and HAV in the upper left thigh and PCV13 in the lower left thigh). Participants in the Hib-MenCY and Hib only groups received an injection of either Hib-MenCY-TT or Hib-OMP (as appropriate) in the upper right thigh (Supplementary material 2 contains the description of the study vaccines).

We conducted the study in accordance with ICH Good Clinical Practice guidelines and the Declaration of Helsinki. Each participant’s parent/guardian gave written informed consent prior to enrolment. Institutional Review Boards at participating sites approved the study. The study is registered at www.clinicaltrials.gov (NCT01978093) and a protocol summary is available at http://www.gsk-clinicalstudyregister.com (study 112931). Anonymized individual participant data and study documents can be requested for further research from www.clinicalstudydatarequest.com.

### Study objectives

This study had 5 co-primary objectives that we assessed in a hierarchical manner (Table 2). The study had to meet the statistical criteria for non-inferiority of the immune response to Hib induced by 4 doses of Hib-MenCY-TT compared with 3 doses of Hib-OMP to conclude on the other co-primary objectives, which were tested in a hierarchical manner independently of one another in each study phase. In the primary vaccination phase, the study had to meet the statistical criteria for non-inferiority of a 2-dose primary vaccination course with HRV in the Hib-MenCY compared with the Hib only group to conclude on the non-inferiority of a 3-dose primary vaccination course of PCV13 in the Hib-MenCY compared with the Hib only group. In the booster vaccination phase, the study had to meet the statistical criteria for non-inferiority of a 2-dose vaccination course with HAV in the Hib-MenCY compared with the Hib only group to conclude on the non-inferiority of a 4-dose vaccination course of PCV13 in the Hib-MenCY compared with the Hib only group. Supplementary Figure 1 presents the sequence for evaluating the study objectives.

Secondary immunogenicity objectives included evaluating the following immune responses: anti-PRP antibody concentrations 2 months after the 2nd dose of Hib-OMP (Hib only group), 1 month after the 3rd dose of Hib-MenCY-TT (Hib-MenCY group) and 1 month post-booster dose after Hib-MenCY-TT or Hib-OMP vaccination; hSBA-MenC and hSBA-MenY titers at 1 month after the 3rd dose and 1 month post-booster dose of Hib-MenCY-TT or Hib-OMP; and anti-HAV at 1 month after the first HAV vaccination in both groups.

Safety objectives included evaluating the safety and reactogenicity of the primary doses of Hib-MenCY-TT or Hib-OMP co-administered with PCV13, HRV and DTaP-HBV-IPV and of the booster doses of Hib-MenCY-TT or Hib-OMP co-administered with HAV and PCV13.

### Immunogenicity assessment

We collected approximately 5 mL of blood from participants at the timepoints specified in Figure 5. We measured anti-HRV, -HAV and -PRP antibody concentrations with in-house enzyme-linked immunosorbent assays (ELISA) at GSK Clinical Laboratory Sciences, Belgium. Anti-HRV IgA concentrations of 20 unit/mL, anti-HAV antibody concentrations of 15 mIU/mL and anti-PRP antibody concentrations of 1 µg/mL were indicative of seroprotection.^18-21^ We measured serum bactericidal antibody titers against MenC and MenY with a hSBA assay at GSK Clinical Laboratory Sciences, Belgium. As previously established for MenC and later extended to MenY, we used hSBA titers above 1:8 to define protection.^22,23^ The Institute of Child Health (London) performed the ELISA to assess antibody concentrations against pneumococcal serotypes 1, 3, 4, 5, 6A, 6B, 7F, 9V, 14, 18C, 19A, 19F and 23F. We used antibody concentrations of 0.35 µg/mL to define protection against the 13 vaccine pneumococcal serotypes.^24,25^

### Safety and reactogenicity assessment

After each vaccination, we collected solicited local (pain, redness and swelling at injection site) and general (drowsiness, fever, irritability and loss of appetite) symptoms on diary cards during a 4-day follow-up period and unsolicited AEs during a 31-day follow-up period. We graded all solicited symptoms and unsolicited AEs by intensity from 1 (mild) to 3 (severe). We defined a SAE as any untoward medical occurrence that resulted in death, was life-threatening, required hospitalization or prolonged existing hospitalization, or resulted in disability or incapacity. We recorded SAEs during the entire study period. We considered all solicited local (injection site) symptoms as causally related to vaccination. The investigator assessed the causality of all other AEs.

### Statistical analyses

Considering a drop-out rate of 25%, we calculated a total sample size of 600 participants (300 per group) to ensure availability of 450 participants for the assessment of HRV and PCV13 in the primary vaccination phase of the study. Considering an additional drop-out rate of 30% for participants completing the primary vaccination phase of the study, 600 initial participants ensured availability of 315 participants for the assessment of Hib, PCV13 and HAV in the booster vaccination phase. To maintain the type I error below 2.5% and to conclude independently on the co-primary objectives of the primary and booster vaccination phases, the first co-primary objective had to be achieved and we used a Bonferroni correction to test the other co-primary objectives (1.25% one-sided for the primary vaccination phase and 1.25% one-sided for the booster vaccination phase). The power of the study to meet the first co-primary objective was ≥ 99.9%, and the overall power of the study was at least 89.5%.

We randomized the infants to the Hib-MenCY and Hib only groups by using an internet-based randomization system at the investigator site. We stratified the randomization within each sub-cohort and used the center as minimization factor. We conducted the safety analyses on the primary and booster TVCs. We performed the analyses of immunogenicity on the primary, booster and HAV ATP immunogenicity cohorts, including all evaluable participants who had assay results for antibodies against at least one antigen for the blood samples taken. Additional immunogenicity analyses were performed on the primary and booster TVCs.

Table 2 contains the statistical non-inferiority criteria for the co-primary objectives. For each treatment group and for each antigen, we assessed seroprotection rates and GMTs/GMCs with exact 95% CIs. We computed antibody GMCs/GMTs by taking the anti-log of the means of the log-transformed antibody titers/concentrations. We obtained the 95% CIs for the GMTs/GMCs by exponential-transformation for the 95% CIs for the means of log transformed concentrations/titers. We obtained the group GMC/GMT ratios using an ANOVA model on the logarithm10 transformation of the concentrations/titers. The ANOVA model included the vaccine group and the blood sample sub-cohort as fixed effect.

MenHibrix, Rotarix, Havrix are trademarks of the GSK group of companies. Prevenar 13/Prevnar 13 is a trademark of Pfizer Inc.

## Abbreviations

ATP: according-to-protocol
AE: adverse event
CI: confidence interval
DTaP-HBV-IPV: diphtheria–tetanus–acellular pertussis–hepatitis B–inactivated poliovirus vaccine
ELISA: enzyme-linked immunosorbent assay
GMC: geometric mean concentration
GMT: geometric mean titer
Hib: *Haemophilus influenzae* type b
Hib-MenCY-TT: combined *Haemophilus influenzae* type b–*Neisseria meningitidis* serogroups C and Y–tetanus toxoid conjugate vaccine
HRV: human rotavirus vaccine
HAV: hepatitis A vaccine
Hib-OMP: Hib vaccine conjugated to *Neisseria meningitidis* outer membrane protein complex
Hsba: human serum bactericidal assay
LL: lower limit; MenB, *Neisseria meningitidis* serogroup B
MenC: *Neisseria meningitidis* serogroup C
MenY: *Neisseria meningitidis* serogroup Y
TT: tetanus toxoid
PCV13: 13-valent pneumococcal conjugate vaccine
PRP: polyribosylribitol phosphate
TVC: total vaccinated cohort
SAE: serious adverse event
US: United States

## Trial registration

NCT01978093 (www.clinicaltrials.gov)
