# Supplementary material for: Immunogenicity and safety of the Haemophilus influenzae type b and Neisseria meningitidis serogroups C and Y-tetanus toxoid conjugate vaccine co-administered with human rotavirus, hepatitis A and 13-valent pneumococcal conjugate vaccines: results from a phase III, randomized, multicenter study in infants
Source: Hum Vaccin Immunother. 2018 Oct 5;15(2):327–38. doi: 10.1080/21645515.2018.1526586 (PMC6422469; doi:10.1080/21645515.2018.1526586)
Supplement: Supplemental Material [file khvi-15-02-1526586-s001.docx]

# Supplemental online material

**Supplementary material 1.** List of inclusion/exclusion criteria

## Inclusion criteria for enrolment

All participants must satisfy ALL the following criteria at study entry:

- Participants’ parent(s)/Legally Acceptable Representative(s) (LAR[s]) who, in the opinion of the investigator, can and will comply, with the requirements of the protocol (*e.g.*, completion of the diary cards, return for follow-up visits).
- A male or female between, and including, 6 and 12 weeks of age at the time of the first vaccination.
- Written informed consent obtained from the parent(s)/LAR(s) of the participant.
- Healthy participants as established by medical history and clinical examination before entering into the study.
- Born full-term (i.e. born after a gestation period of at least 37 weeks inclusive).
- Infants who have not received a previous dose of hepatitis B vaccine or those who have received only 1 dose of hepatitis B vaccine administered at least 30 days prior to enrolment.

## Exclusion criteria for enrolment

The following criteria should be checked at the time of study entry. If ANY exclusion criterion applies, the participant must not be included in the study:

- Child in care
- Use of any investigational or non-registered product (drug or vaccine) other than the study vaccine(s) within 30 days preceding the dose of study vaccine or planned use during the study period.
- Chronic administration (defined as more than 14 days in total) of immunosuppressants or other immune-modifying drugs since birth prior to the first vaccine dose. For corticosteroids, this will mean prednisone ≥ 0.5 mg/kg/day, or equivalent. Inhaled and topical steroids are allowed.
- Previous vaccination against *Neisseria meningitidis*, *Haemophilus influenzae* type b, diphtheria, tetanus, pertussis, rotavirus, pneumococcus, hepatitis A and/or poliovirus; more than one previous dose of hepatitis B vaccine.
- Planned administration/administration of a vaccine not foreseen by the study protocol in the period starting 30 days before and ending 30 days after the dose of vaccines. Participants may receive inactivated influenza vaccine or pandemic influenza vaccines any time during the study according to the national recommendation. Measles, mumps, rubella and varicella vaccination are allowed 30 days before or 30 days after the final vaccination of Hib-MenCY-TT or *PedvaxHIB* (Hib-OMP).
- History of *Neisseria meningitidis*, *Haemophilus influenzae* type b, diphtheria, tetanus, pertussis, pneumococcus, hepatitis B, hepatitis A, rotavirus, and/or poliovirus disease.
- Any confirmed or suspected immunosuppressive or immunodeficient condition based on medical history and physical examination (no laboratory testing required).
- History of allergic disease or reactions likely to be exacerbated by any component of the vaccines, including dry natural latex rubber. Hypersensitivity to any component of the vaccines, including gelatin or neomycin.
- Major congenital defects or serious chronic illnesses.
- History of any neurologic disorders or seizures. A single, simple febrile seizure is allowed.
- Participants with history of intussusceptions or uncorrected congenital malformation of the gastrointestinal tract that would predispose for intussusceptions.
- Acute disease and/or fever at the time of enrolment.
- Fever is defined as temperature ≥38.0°C/100.4°F by any method. The preferred route for recording temperature in primary phase will be rectal and axillary in booster phase.
- Participants with minor illness (such as mild diarrhoea, mild upper respiratory infection) without fever may, be enrolled at the discretion of the investigator.
- Administration of immunoglobulins and/or blood products since birth or planned administration during the study period.

## Supplementary material 2. Study vaccines

One 0.5 mL dose of Hib-MenCY-TT contained 2.5 µg of purified capsular polysaccharide polyribosylribitol phosphate (PRP) and 5 μg each of MenC and MenY polysaccharides conjugated to tetanus toxoid (TT; ~20 µg) and 150 mM NaCl. Each 0.5 mL dose of the Hib-OMP vaccine contained 7.5 µg of PRP, 125 µg of *N. meningitidis* outer membrane protein complex and 225 µg of Al_2_(PO_4_) (OH) (SO_4_). A 1 mL suspension of HRV contained 10^6.5^ cell culture infectious dose 50% (CCID_50_) of HRV strain RIX4144 and 60 mg of CaCO_3_ diluent. The HAV vaccine consisted of 720 ELISA units (ELU) hepatitis A virus strain HM175 and 250 µg Al(OH)_3_. One dose of PCV13 contained 2.2 µg each of pneumococcal capsular polysaccharides of serotypes 1, 3, 4, 5, 6A, 7F, 9V, 14, 18C, 19A, 19F, 23F and 4.4 µg of pneumococcal capsular polysaccharide serotype 6B individually conjugated to diphtheria toxoid CRM_197_ protein and 125 µg AlPO_4_. Each 0.5 mL dose of DTaP-HBV-IPV vaccine contained ≥30 international units (IU) of diphtheria toxoid, ≥40 IU of TT, 25 µg of pertussis toxin (PT), 25 µg of filamentous hemagglutinin (FHA), 8 µg of pertactin (PRN), 10 µg of hepatis B surface antigen (HBsAg), 40 D-antigen Units (DU) of inactivated poliovirus type 1 (Mahoney strain), 8 DU of inactivated poliovirus type 2 (MEF-1 strain), and 32 DU of inactivated poliovirus type 3 (Saukett strain), and 700 µg of Al_2_(PO_4_) (OH) (SO_4_).

**Supplementary Table 1.** Summary of demographic characteristics (booster ATP immunogenicity cohorts)

| **Characteristics** | **Parameters** | **Hib-MenCY group** | **Hib only group** |
| --- | --- | --- | --- |
| **Booster vaccination phase, ATP immunogenicity cohort** | | N=223 | N=218 |
| Age at first dose (months) | Mean (SD) | 12.5 (0.8) | 12.6 (0.8) |
| Gender | Female, n (%) | 111 (49.8) | 102 (46.8) |
| Race | White-Caucasian / European Heritage, n (%) | 159 (71.3) | 161 (73.9) |
|  | African Heritage / African American, n (%) | 18 (8.1) | 11 (5.0) |
|  | American Indian or Alaskan Native, n (%) | 8 (3.6) | 9 (4.1) |
|  | Asian - Central/South Asian Heritage, n (%) | 3 (1.3) | 3 (1.4) |
|  | Asian - East Asian Heritage, n (%) | 2 (0.9) | 0 (0.0) |
|  | Asian - South East Asian Heritage, n (%) | 6 (2.7) | 8 (3.7) |
|  | Native Hawaiian or other Pacific Islander, n (%) | 2 (0.9) | 1 (0.5) |
|  | White - Arabic / North African Heritage, n (%) | 1 (0.4) | 1 (0.5) |
|  | Other, n (%) | 24 (10.8) | 24 (11.0) |
| Hepatitis B vaccination at birth | Yes, n (%) | 214 (96.0) | 210 (96.3) |
| **Booster vaccination phase, HAV ATP immunogenicity cohort** | | N=129 | N=124 |
| Age at first dose (months) | Mean (SD) | 12.6 (0.7) | 12.7 (0.8) |
| Age at second dose (months) | Mean (SD) | 18.8 (0.8) | 18.8 (0.9) |
| Gender | Female, n (%) | 66 (51.2) | 58 (46.8) |
| Race | White-Caucasian / European Heritage, n (%) | 91 (70.5) | 85 (68.5) |
|  | African Heritage / African American, n (%) | 6 (4.7) | 6 (4.8) |
|  | American Indian or Alaskan Native, n (%) | 6 (4.7) | 6 (4.8) |
|  | Asian - Central/South Asian Heritage, n (%) | 1 (0.8) | 1 (0.8) |
|  | Asian - East Asian Heritage, n (%) | 2 (1.6) | 0 (0.0) |
|  | Asian - Japanese Heritage, n (%) | 1 (0.8) | 0 (0.0) |
|  | Asian - South East Asian Heritage, n (%) | 6 (4.7) | 8 (6.5) |
|  | Native Hawaiian or other Pacific Islander, n (%) | 1 (0.8) | 1 (0.8) |
|  | Other | 15 (11.6) | 17 (13.7) |
| Hepatitis B vaccination at birth | Yes, n (%) | 121 (93.8) | 121 (97.6) |

Footnote: N, number of participants; SD, standard deviation, n (%), number (percentage) of participants in a given category, ATP, according-to-protocol

**Supplementary Table 2.** Summary of the co-primary objectives (primary and booster TVCs)

| **Evaluation** | |  | **Results** |  |
| --- | --- | --- | --- | --- |
| **Objective** | **Statistical criterion** |  | **Assessed outcome** | **Value (95% CI)** |
| 1. To demonstrate the non-inferiority of a 4-dose vaccination course with Hib-MenCY-TT compared with a 3-dose vaccination course with Hib-OMP, each co-administered with PCV13 and HAV, in terms of anti-PRP antibody concentration ≥1.0 µg/mL.^a^ | One month post-dose 4: LL of standardized asymptotic 95% CI for the difference in % of participants with anti-PRP antibody concentration ≥1.0 µg/mL between two groups ≥-10%. |  | Difference between groups (anti-PRP antibody concentration):  Hib-MenCY minus Hib only group | 0.35 (**-2.74**; 3.48) |
| **Primary vaccination phase** | | | | **Value (97.5% CI)** |
| 2. To demonstrate the non-inferiority of a 2-dose primary vaccination course with HRV co-administered with Hib-MenCY-TT, DTaP-HBV-IPV and PCV13 compared with that of HRV co-administered with Hib-OMP, DTaP-HBV-IPV and PCV13 in terms of anti-HRV IgA GMCs. | Two months post-dose 2: LL of 97.5% CI of anti-HRV IgA GMC ratio ≥0.5. |  | Anti-HRV IgA GMC ratio between groups:  Hib-MenCY group/Hib only group | 1.25 (**0.80**; 1.96) |
| 3. To demonstrate the non-inferiority of a 3-dose primary vaccination course of PCV13 co-administered with Hib-MenCY-TT, HRV and DTaP-HBV-IPV compared with that of PCV13 co-administered with Hib-OMP, HRV and DTaP-HBV-IPV in terms of anti-pneumococcal antibody GMCs. | One month post-dose 3: LL of 97.5% CI of anti-pneumococcal serotypes 1, 3, 4, 5, 6A, 6B, 7F, 9V, 14, 18C, 19A, 19F and 23F antibody GMC ratio ≥ 0.5. |  | Anti-pneumococcal antibody GMC ratio between groups:  Hib-MenCY group/Hib only group | anti-1, 1.16 (**0.94**; 1.43)  anti-3, 1.16 (**0.95**; 1.43)  anti-4, 1.07 (**0.89**; 1.28)  anti-5, 1.16 (**0.94**; 1.44)  anti-6A 1.24 (**0.99**; 1.54)  anti-6B 1.15 (**0.88**; 1.52)  anit-7F, 1.10 (**0.92**; 1.33)  anti-9V, 1.20 (**0.97**; 1.48)  anti-14, 1.17 (**0.91**; 1.49)  anti-18C, 1.22 (**1.01**; 1.48)  anti-19A, 1.14 (**0.93**, 1.40)  anti-19F, 1.06 (**0.89**; 1.27)  anti-23F, 1.13 (**0.88**; 1.45) |
| **Booster vaccination phase** |  |  |  |  |
| 4. To demonstrate the non-inferiority of a 2-dose vaccination course of HAV when the first dose is co-administered with Hib-MenCY-TT and PCV13 compared with that of HAV when the first dose is co-administered with Hib-OMP and PCV13 in terms of anti-HAV antibody concentration ≥15 mIU/mL. | One month after the second HAV vaccination: LL of standardized asymptotic 97.5% CI for the difference in % of participants with antibody concentration ≥15 mIU/mL between two groups ≥-10%. |  | Difference between groups (anti-HAV antibody concentration):  Hib-MenCY minus Hib only group | 0.00 (**-3.34**; 3.45) |
| 5. To demonstrate the non-inferiority of a 4-dose vaccination course of PCV13 co-administered with Hib-MenCY-TT and HAV compared with that of PCV13 co-administered with Hib-OMP and HAV in terms of anti-pneumococcal antibody GMCs. | One month post-dose 4: LL of 97.5% CI of anti-pneumococcal serotypes 1, 3, 4, 5, 6A, 6B, 7F, 9V, 14, 18C, 19A, 19F and 23F antibody GMC ratio ≥ 0.5. |  | Anti-pneumococcal antibody GMC ratio between groups:  Hib-MenCY group/Hib only group | anti-1, 1.25 (**1.05**; 1.49)  anti-3, 1.00 (**0.82**; 1.21)  anti-4, 1.11 (**0.94**; 1.32)  anti-5, 1.08 (**0.90**; 1.30)  anti-6A 1.19 (**1.00**; 1.41)  anti-6B 1.14 (**0.95**; 1.36)  anit-7F, 1.10 (**0.94**; 1.29)  anti-9V, 1.11 (**0.94**; 1.31)  anti-14, 1.15 (**0.96**; 1.38)  anti-18C, 1.17 (**0.99**; 1.37)  anti-19A, 1.11 (**0.92**, 1.34)  anti-19F, 1.15 (**0.97**; 1.36)  anti-23F, 1.21 (**1.00**; 1.47) |

Footnote: CI, confidence interval; GMC, geometric mean concentration; HAV, hepatitis A vaccine; IgA, immunoglobulin A; LL, lower limit; PRP, polyribosylribitol phosphate; TVC, total vaccinated cohort

**Supplementary Table 3.** Immune responses to the study vaccines in the primary vaccination phase (primary ATP immunogenicity cohort)

| **Antibody** | **Threshold** |  | **Hib-MenCY group** | | |  | | **Hib only group** | | |
| --- | --- | --- | --- | --- | --- | --- | --- | --- | --- | --- |
|  |  |  | **N** | **% (95% CI)** | **GMC (95% CI)** |  | **N** | **% (95% CI)** | **GMC (95% CI)** |  |
| **One month post-dose 3 (Hib-MenCY Group) and two months post-dose 2 (Hib only group)** | | | | | | | | | | |
| Anti-PRP | ≥ 1 µg/mL |  | 167 | 94.0 (89.3; 97.1) | 8.414 (7.070; 10.014) |  | 165 | 91.5 (86.2; 95.3) | 11.053 (8.740; 13.979) |  |
| **Two months post-dose 2** | | | | | | | | | | |
| Anti-HRV IgA | ≥ 20 U/mL |  | 155 | 81.3 (74.2; 87.1) | 138.9 (104.0; 185.5) |  | 161 | 80.1 (73.1; 86.0) | 115.0 (87.5; 151.0) |  |
| **One month post-dose 3** | | | | | | | | | | |
| Anti-1 | ≥ 0.35 µg/mL |  | 156 | 96.8 (92.7; 99.0) | 1.49 (1.30; 1.70) |  | 158 | 93.7 (88.7; 96.9) | 1.26 (1.10; 1.44) |  |
| Anti-3 | ≥ 0.35 µg/mL |  | 149 | 69.8 (61.7; 77.0) | 0.55 (0.48; 0.63) |  | 150 | 69.3 (61.3; 76.6) | 0.48 (0.42; 0.55) |  |
| Anti-4 | ≥ 0.35 µg/mL |  | 156 | 91.0 (85.4; 95.0) | 0.81 (0.72; 0.90) |  | 158 | 84.8 (78.2; 90.0) | 0.74 (0.66; 0.84) |  |
| Anti-5 | ≥ 0.35 µg/mL |  | 156 | 91.0 (85.4; 95.0) | 0.80 (0.71; 0.91) |  | 158 | 80.4 (73.3; 86.3) | 0.68 (0.59; 0.78) |  |
| Anti-6A | ≥ 0.35 µg/mL |  | 156 | 98.1 (94.5; 99.6) | 1.76 (1.55; 2.00) |  | 158 | 91.8 (86.3; 95.5) | 1.37 (1.18; 1.60) |  |
| Anti-6B | ≥ 0.35 µg/mL |  | 154 | 83.8 (77.0; 89.2) | 1.00 (0.85; 1.18) |  | 158 | 80.4 (73.3; 86.3) | 0.87 (0.73; 1.05) |  |
| Anti-7F | ≥ 0.35 µg/mL |  | 156 | 100 (97.7; 100) | 2.59 (2.29; 2.93) |  | 158 | 100 (97.7; 100) | 2.36 (2.10; 2.65) |  |
| Anti-9V | ≥ 0.35 µg/mL |  | 156 | 83.3 (76.5; 88.8) | 0.78 (0.69; 0.89) |  | 157 | 76.4 (69.0; 82.8) | 0.63 (0.55; 0.73) |  |
| Anti-14 | ≥ 0.35 µg/mL |  | 156 | 99.4 (96.5; 100) | 4.77 (4.13; 5.52) |  | 156 | 97.4 (93.6; 99.3) | 4.16 (3.50; 4.94) |  |
| Anti-18C | ≥ 0.35 µg/mL |  | 156 | 87.2 (80.9; 92.0) | 0.91 (0.81; 1.03) |  | 158 | 82.3 (75.4; 87.9) | 0.74 (0.65; 0.84) |  |
| Anti-19A | ≥ 0.35 µg/mL |  | 156 | 97.4 (93.6; 99.3) | 1.31 (1.17; 1.48) |  | 158 | 90.5 (84.8; 94.6) | 1.13 (0.98; 1.31) |  |
| Anti-19F | ≥ 0.35 µg/mL |  | 155 | 98.7 (95.4; 99.8) | 2.25 (2.02; 2.50) |  | 158 | 100 (97.7; 100) | 2.10 (1.87; 2.37) |  |
| Anti23F | ≥ 0.35 µg/mL |  | 156 | 83.3 (76.5; 88.8) | 0.94 (0.80; 1.10) |  | 157 | 77.1 (69.7; 83.4) | 0.80 (0.67; 0.94) |  |
| **One month post-dose 3** | | | | | | | | | | |
| MenC | ≥ 1:8 |  | 144 | 100 (97.5; 100) | 807.3 (659.2; 988.6) |  | 141 | 1.4 (0.2; 5.0) | 2.1 (2.0; 2.3) |  |
| MenY | ≥ 1:8 |  | 130 | 97.7 (93.4; 99.5) | 510.9 (405.7; 643.3) |  | 150 | 100 (97.6; 100) | 550.2 (474.4; 638.1) |  |

Footnote: ATP, according-to-protocol; CI, confidence interval; GMC/GMT, geometric mean concentration/titer; HRV, human rotavirus vaccine; IgA, immunoglobulin A; MenC and Y, meningococcal serogroups C and Y; PRP, polyribosylribitol phosphate

**Supplementary Table 4.** Immune responses to the study vaccine in the booster vaccination phase (booster ATP immunogenicity cohort and HAV ATP immunogenicity cohort)

| **Antibody** | **Threshold** |  | **Hib-MenCY group** | | | | |  | **Hib only group** | | | | |
| --- | --- | --- | --- | --- | --- | --- | --- | --- | --- | --- | --- | --- | --- |
|  |  |  | **N** |  | **% (95% CI)** |  | **GMC/GMT (95% CI)** |  | **N** |  | **% (95% CI)** |  | **GMC/GMT (95% CI)** |
| **One month post-dose 4 (booster ATP immunogenicity cohort)** | | | | | | | | | | | | | |
| Anti-PRP | ≥ 1 µg/mL |  | 223 |  | 98.2 (95.5; 99.5) |  | 28.090 (24.012; 32.862) |  | 218 |  | 97.2 (94.1; 99.0) |  | 20.869 (17.799; 24.468) |
| Anti-1 | ≥ 0.35 µg/mL |  | 216 |  | 97.7 (94.7; 99.2) |  | 2.00 (1.78; 2.25) |  | 205 |  | 97.1 (93.7; 98.9) |  | 1.60 (1.43; 1.79) |
| Anti-3 | ≥ 0.35 µg/mL |  | 169 |  | 71.6 (64.2; 78.3) |  | 0.52 (0.46; 0.58) |  | 167 |  | 69.5 (61.9; 76.3) |  | 0.51 (0.44; 0.59) |
| Anti-4 | ≥ 0.35 µg/mL |  | 216 |  | 97.7 (94.7; 99.2) |  | 1.36 (1.23; 1.50) |  | 205 |  | 94.1 (90.0; 96.9) |  | 1.24 (1.10; 1.39) |
| Anti-5 | ≥ 0.35 µg/mL |  | 215 |  | 99.1 (96.7; 99.9) |  | 2.36 (2.10; 2.64) |  | 205 |  | 98.0 (95.1; 99.5) |  | 2.23 (1.97; 2.52) |
| Anti-6A | ≥ 0.35 µg/mL |  | 216 |  | 100 (98.3; 100) |  | 6.80 (6.10; 7.57) |  | 205 |  | 100 (98.2; 100) |  | 5.63 (5.05; 6.26) |
| Anti-6B | ≥ 0.35 µg/mL |  | 215 |  | 100 (98.3; 100) |  | 5.57 (4.97; 6.24) |  | 205 |  | 100 (98.2; 100) |  | 4.94 (4.40; 5.55) |
| Anti-7F | ≥ 0.35 µg/mL |  | 216 |  | 100 (98.3; 100) |  | 4.16 (3.76; 4.61) |  | 205 |  | 99.5 (97.3; 100) |  | 3.81 (3.45; 4.21) |
| Anti-9V | ≥ 0.35 µg/mL |  | 215 |  | 97.7 (94.7; 99.2) |  | 1.38 (1.25; 1.53) |  | 205 |  | 96.6 (93.1; 98.6) |  | 1.24 (1.11; 1.39) |
| Anti-14 | ≥ 0.35 µg/mL |  | 216 |  | 99.5 (97.4; 100) |  | 7.14 (6.32; 8.07) |  | 205 |  | 100 (98.2; 100) |  | 6.13 (5.48; 6.86) |
| Anti-18C | ≥ 0.35 µg/mL |  | 216 |  | 98.6 (96.0; 99.7) |  | 1.62 (1.46; 1.80) |  | 204 |  | 97.5 (94.4; 99.2) |  | 1.42 (1.28; 1.57) |
| Anti-19A | ≥ 0.35 µg/mL |  | 216 |  | 100 (98.3; 100) |  | 5.47 (4.87; 6.14) |  | 204 |  | 100 (98.2; 100) |  | 5.03 (4.47; 5.64) |
| Anti-19F | ≥ 0.35 µg/mL |  | 215 |  | 100 (98.3; 100) |  | 6.23 (5.61; 6.92) |  | 205 |  | 100 (98.2; 100) |  | 5.54 (4.97; 6.18) |
| Anti23F | ≥ 0.35 µg/mL |  | 216 |  | 98.1 (95.3; 99.5) |  | 3.28 (2.90; 3.71) |  | 205 |  | 100 (98.2; 100) |  | 2.68 (2.37; 3.05) |
| MenC | ≥ 1:8 |  | 215 |  | 99.1 (96.7; 99.9) |  | 2566.2 (2046.3; 3218.1) |  | 168 |  | 0.6 (0.0; 3.3) |  | 2.0 (2.0; 2.1) |
| MenY | ≥ 1:8 |  | 198 |  | 98.5 (95.6; 99.7) |  | 2761.4 (2274.2; 3353.1) |  | 187 |  | 100 (98.0; 100) |  | 2728.2 (2412.7; 3085.0) |
| **One month post-dose 1 of HAV vaccine (****booster ATP immunogenicity cohort)** | | | | | | | | | | | | | |
| HAV | ≥ 15 mIU/mL |  | 182 |  | 85.2 (79.2; 90.0) |  | 44.8 (38.3; 52.5) |  | 168 |  | 89.3 (83.6; 93.5) |  | 47.3 (40.9; 54.8) |
| **One month post-dose 2 of HAV vaccine (HAV immunogenicity cohort)** | | | | | | | | | | | | | |
| HAV | ≥ 15 mIU/mL |  | 129 |  | 100 (97.2; 100) |  | 1590.7 (1312.7; 1927.5) |  | 124 |  | 100 (97.1; 100) |  | 1390.6 (1147.8; 1684.6) |

Footnote: ATP, according-to-protocol; CI, confidence interval; GMC/GMT, geometric mean concentration/titer; HAV, hepatitis A virus; MenC and Y, meningococcal serogroups C and Y; PRP, polyribosylribitol phosphate

**Supplementary Figure 1.** Sequence for evaluating the study objectives to control the overall type I error below 2.5% (one-sided)


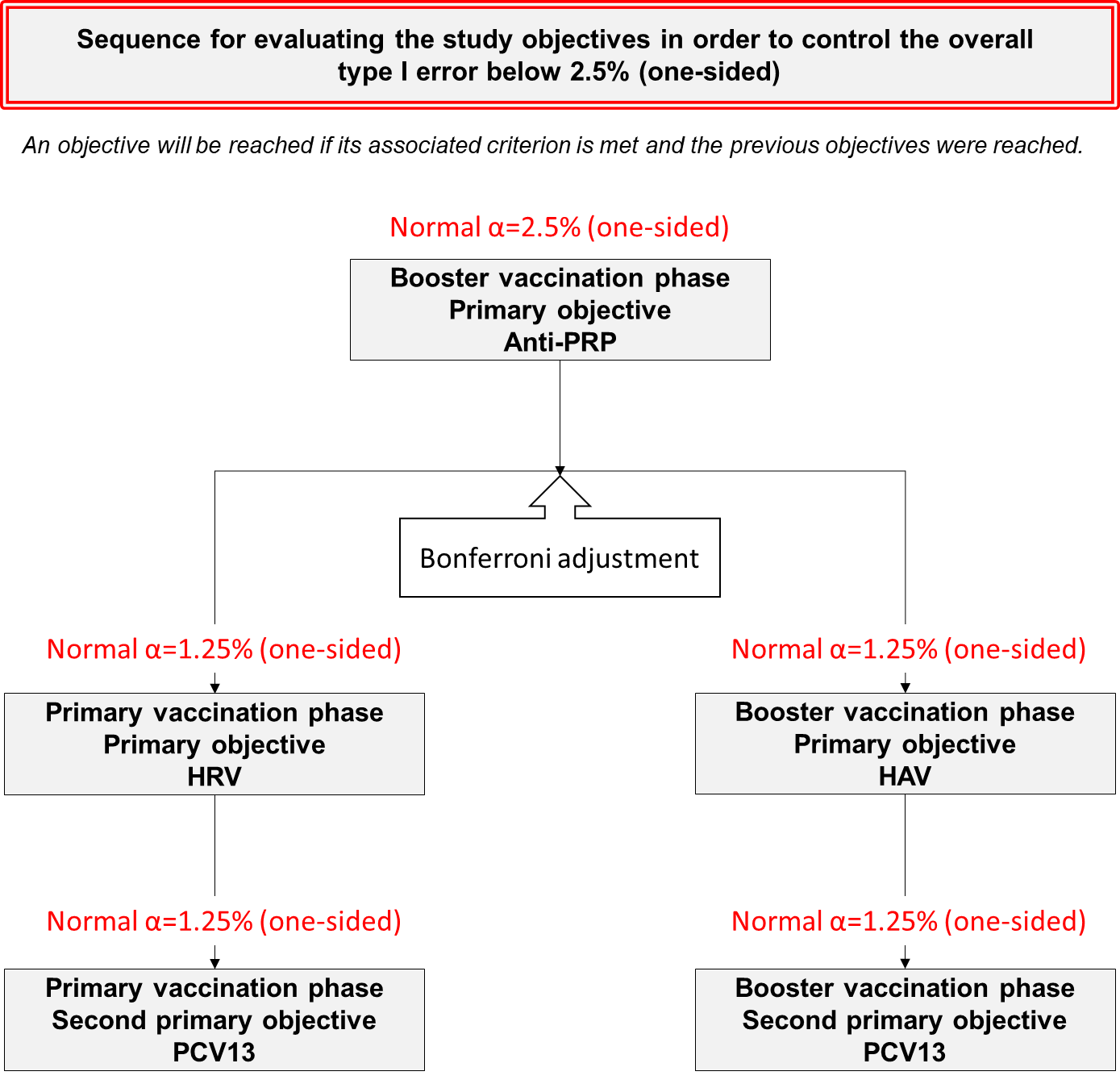


Footnote: α, type I error; HAV, hepatitis A vaccine; HRV, human rotavirus vaccine; PCV13, 13-valent pneumococcal conjugate vaccine; PRP, polyribosylribitol phosphate.
